# Supplementary material for: Radiomics Features in Predicting Human Papillomavirus Status in Oropharyngeal Squamous Cell Carcinoma: A Systematic Review, Quality Appraisal, and Meta-Analysis
Source: Diagnostics (Basel). 2024 Mar 29;14(7):737. doi: 10.3390/diagnostics14070737 (PMC11011663; doi:10.3390/diagnostics14070737)
Supplement: Supplementary file 1 [file diagnostics-14-00737-s001.zip › diagnostics-2808443-supplementary.pdf]

Supplementary material:

Table S1. Quality of the included studies based on QUADAS-2 tool

Study	RISK OF BIAS				APPLICABILITY CONCERNS		
	PATIENT SELECTION	INDEX TEST	REFERENCE STANDARD	FLOW AND TIMING	PATIENT SELECTION	INDEX TEST	REFERENCE STANDARD
Boot et al.	☺	☺	☺	☺	☺	☺	☺
Bos et al.	?	?	☺	☺	☺	?	☺
Bagher et al	?	?	?	☺	?	☺	☺
Park et al	☹	?	?	☺	☺	☺	☺
Wenbing	☺	☺	☺	☺	?	☺	☺
Marzi et al.	?	☺	?	☺	?	☺	☺
Sohn et al.	?	?	☺	☺	?	☺	☺
Bagher et al	?	☺	☺	☺	☺	☺	☺
Song et al.	☹	?	☺	☺	☹	☺	☺
Ebadian	?	?	☺	?	?	☺	☺
Bogowicz	?	?	☺	?	?	☺	☺
Bos et al.	?	?	☺	☹	☺	☺	☺
Choi et al.	?	☺	☺	☺	?	☺	☺
Haider etal	☺	☺	☺	☺	☺	☺	☺
Ren et al.	☺	☺	☺	☺	☺	☺	☺
Suh et al	☺	☺	☺	☺	☺	☺	☺
Wenbing	☺	☺	☺	☺	?	☺	☺
Marzi et al.	?	☺	?	☺	?	☺	☺
Sohn et al.	?	?	☺	☺	?	☺	☺
Mungai et al	?	?	☺	☺	?	?	☺
Huang et al.	☺	☺	☺	☺	?	☺	☺
Lee et al.	☹	?	?	?	☹	☺	?
Leijenaar	?	?	☺	?	?	☺	☺
Ranjbar etal	?	?	☺	☹	☺	☹	☺
Ravanelli	☺	☺	?	☺	☺	☺	☺
Bogowicz	☹	?	☺	☺	☹	?	☺
Bagher et al	?	?	?	☺	?	☺	☺
Park et al	☹	?	?	☺	☺	☺	☺
Wenbing	☺	☺	☺	☺	?	☺	☺
Marzi et al.	?	☺	?	☺	?	☺	☺
Sohn et al.	?	?	☺	☺	?	☺	☺
Yu et al.	?	☺	☺	☺	?	☺	☺
Fujita et al.	☹	?	☺	☺	☹	☺	☺
Buch et al.	☹	?	☺	?	☺	☹	☺

Table S2. Radiomics Quality Score of the included studies

Study name	Image protocol quality	Multiple segmentation	Phantom study	: Multiple time points	Feature reduction	Multivariable analysis	Biological correlates	Cut-off analysis	Discrimination statistics	Calibration statistics	Prospective design	Validation	Comparison to "gold standard"	Potential clinical application	Cost-efectiveness analysis	Open science and data	SUM
Boot et al.	1	0	0	0	3	1	0	0	1	0	0	2	1	0	0	1	10
Bos et al.	1	0	0	0	3	1	0	1	1	0	0	2	1	0	0	0	10
Bagher-Ebadian 2022	1	0	0	0	3	1	0	0	1	0	0	2	1	0	0	0	9
Park 2022	1	1	0	0	3	0	0	0	1	0	0	2	1	0	0	0	9
Wenbing 2022	1	1	0	0	3	0	0	1	2	0	0	4	1	0	0	1	14
Marzi 2022	0	0	0	0	3	1	0	1	1	0	0	2	1	0	0	0	9
Sohn 2021	1	0	0	0	3	0	0	0	1	0	0	2	1	0	0	0	8
Bagher Ebadian 2021	0	0	0	0	3	0	0	0	1	0	0	2	1	0	0	0	7
Song 2021	1	1	0	0	3	1	1	0	2	2	0	3	1	1	0	0	16
Ebadian 2020	0	0	0	0	3	0	0	0	1	0	0	2	1	0	0	0	7
Bogowicz 2020	1	0	0	0	3	0	0	0	1	0	0	5	1	0	0	0	11
Bos et al, 2020	1	0	0	0	3	1	1	1	1	0	0	-5	1	0	0	0	4
Choi 2020	1	1	0	0	3	1	1	0	0	0	0	2	1	0	0	0	10

Stefan P Haider 2020	1	0	0	0	3	0	0	0	1	0	0	5	1	0	0	0	11
Ren et al. 2020	1	1	0	0	3	0	0	0	1	0	0	-5	1	0	0	0	2
Suh et al. 2020	1	0	0	0	3	0	0	0	1	2	0	-5	1	0	0	0	3
Mungai et al. 2020	1	0	0	0	-3	0	0	0	0	0	0	-5	1	0	0	0	0
Huang et al. 2019	1	0	0	0	3	1	1	0	1	0	0	3	1	0	0	1	12
Young lee et al. 2019	1	0	0	0	-3	0	0	0	0	0	0	-5	1	0	0	0	0
Leijenaar 2018 et al.	1	0	0	0	3	0	0	0	1	0	0	3	1	0	0	1	10
Ranjbar et al. 2018	1	0	0	0	3	0	0	0	1	0	0	-5	1	0	0	1	2
Ravanelli et al. 2018	1	0	0	0	-3	1	1	0	1	0	0	-5	1	0	0	0	0
Bogowicz et a 2017	1	0	0	0	3	0	0	0	1	0	0	2	1	0	0	0	8
Yu et al. 2017	1	1	0	0	3	0	0	0	1	0	0	-5	1	0	0	0	2
Fujita et al. 2016	1	0	0	0	-3	0	0	0	0	0	0	-5	1	0	0	0	0
Buch et al. 2015	1	0	0	0	-3	0	0	0	0	0	0	-5	1	0	0	0	0
